# Medical Items

**Published:** 1917-03

**Authors:** 


### MEDICAL ITEMS

### PITUITRIN IN TWO STRENGTHS.

The pituitary extract formerly known as "Pituitrin" and supplied in ampoules will hereafter be designated "Pituitrin O." A second preparation of the pituitary gland, bearing the title of " "Pituitrin S," is now announced. Pituitrin S is approximately twice the strength of Pituitrin O. Both products are manufactured by Parke, Davis & Co.

Pituitrin O (obstetrical) is intended primarily for use in delayed parturition due to uterine inertia. It has been called "an indispensable item in the armamentarium of the obstetrician."

Pituitrin S (surgical) is indicated specifically in the treatment of post-operative intestinal paresis, vesical atony, hemorrhage, shock, etc. It is of the utmost utility in the hands of the surgeon and internist.

Pituitrin O is supplied in ampoules of 1 mil (1 Cc.) and ½ mill (½ Cc.) Pituitrin S is supplied in ampoules of 1 mil (1 Cc.) only.

---

THE INTEROL TREATMENT OF CHRONIC CONSTIPATION IN THE ELDERLY is rational. It fills one want in a harmless and effective way, namely, the want of colonic lubrication.

While lubricating, it also acts beneficially by softening—or rather by keeping soft—the intestinal contents, and by protecting any bare spots in the tract. Finally, it combats the accompanying auto-toxemia by slucing out the feces with their toxins, as is evidenced by the improvement in complexion and in general well-being shown by many of these patients after steadily using INTEROL.

All this is done without enervation to an already weakened organism.

Is there any one thing at the physician's command that will do as much for these patients?

NOTE—Further literature on this subject on request. Van Horn and Sawtell, 15-17 E. 40th St., New York City.

---

### SIMPLIFIED METHOD OF TREATING RABIES.

A survey of the statistics of cases of human rabies shows that it is not known how much or how little of the virus when injected, may result in the production of rabies in untreated cases. This fact makes it imperative that every patient should be given the full benefit of the doubt, and should receive a course which will confer the highest degree of protection. Such a course is the Lilly Intentive Treatment.

The Eli Lilly & Company Rabies Virus is prepared after Harris' (Dr. D. L. Harris, Professor of Hygiene and Preventive Medicine, St. Louis University, School of Medicine) modification of the Pasteur Antirabic Preventive Treatment. The vaccine virus is a standardized powder, the result of desicating the pulverized frozen brains and cords of rabbits dead from fixed virus inoculation.

The advantages claimed for this method are its safety; the high immunizing quality of the vaccine; its economy of time and expense to the patient; the availability of the initial doses for prompt administration and the standardization of dosage.

The Lilly Intensive Treatment consists of fourteen doses (one daily) of the emulsified, powdered virus. The vaccine is marketed in syringe containers and is ready for immediate injection by the physician, who may administer the virus in his office or at the home of the patient. The treatment does not deter the patient from his daily routine.

### CHRONIC CONSTIPATION OF WOMEN

In the treatment of this condition, what the physician may expect INTEROL to do is the following:

(1) It keeps the feces from becoming dried and hard. That is, it keeps them **soft** and **plastic**; (2) and in addition, by **lubricating** them, it (3) enables them to squeeze or slip through angulations, convolutions and constrictions of a crowded gut; (4) at the same time, there is a **protective** action to any raw or abraded spots.

By doing these things, INTEROL relieves fecal pressure and gaseous distention, so that the autotoxic as well as nervous symptoms are likely to be reached.

All these it does effectively and harmlessly. Its use does not prevent the adjunctory use of any orthopaedic, surgical or other procedure that may be indicated. On the contrary, INTEROL itself is more adjunct to such other measures.

INTEROL is unquestionably all that it is claimed to be—a valuable "dietetic accessory." There is no other accessory measure that will better accomplish what Interol does accomplish in cases where it can accomplish it.

DOSAGE is usually a tablespoonful morning and night on an empty stomach, although this varies with the individual peculiarities.

NOTE—Booklet and samples to physicians. Van Horn & Sawtell, 15-17 E. 40th St., New York.

---

### THE AFTERMATH OF WINTER INFECTIONS.

As means of restoring a patient just recovering from a severe bronchial or pulmonary infection and possibly in a fair way to lapse into a chronic condition, Cord. Ext. Ol. Morrhuae Comp. (Hagee) is of more than ordinary worth.

An acute bronchial or pulmonary infection often-times makes the patient highly susceptible to an obstinate and probably serious chronic disorder. In meeting such a condition the national plan is to institute a line of treatment calculated to increase the recuperative powers of the affected tissues, for which purpose Cord. Ext. Ol. Marrhuae Comp. (Hagee) will prove markedly useful. Its palatability is a decided advantage.

### A WINTER INDICATION FOR PAPINE.

For the purpose of securing relief in severe, racking coughs PAPINE (Battle) added to suitable expectorant mixtures is of prompt and marked service. Papine (Battle) is a refined opium product and is indicated in any condition pointing to the need of an opium derivative. It is carefully compounded from the purest of ingredients and offers the prescriber aminimum of therapeutic influence with a minimum of disagreeable effect. For this reason its incorporation in an expectorant mixture, when immediate relief from the highly inflamed bronchial mucosa is demanded, is rational and productive of gratifying results.

Sanmetto is a mild, non-irritating diuretic, which allays urinary irritation and increases urinary secretion. It is thought of in prostatitis, pyelitis, purulent or catarrhal cystitis, irritable condition of the bladder, gonorrhea, enuresis in children, and in fevers where a mild diuretic is desirable to increase the secretion of urine. Sanmetto has been used by thousands of physicians in old men with irritable bladder and difficult urination, and they have found it a very satisfactory medicine. It is safe and harmless, and by its soothing action on the mucous membrane of the bladder, it relieves the irritation, and adds greatly to the comfort of the patient. It increases the flow of urine, lessens the specific gravity, clears up cloudy urine, and relieves undue acidity. In all these ways it is of great benefit to the patient. In enlarged prostate it has done good service by its soothing qualities while reducing the enlargement.

Acute catarrhal infflammation of the upper air passages—coryza, rhinitis, etc., popularly known as catarrh or "a cold," which is prevalent in all parts of the country in all seasons and particularly at this time of the year, while never serious, it renders the victim entirely uncomfortable, and is often quite painful. Usually this condition is an independent affection, but often it is the first sign of the development of another disease; in any event, the severe manifestations call for some agent of relief.

The thin, irritating excretion makes the nostrils sore; there is inflammation of the mucous membrane of the septum with an engorgement of the lower turbinated bodies; the mucous membrane of the tear duct is swollen; pain over the frontal sinuses; slight soreness of the throat and stiffness of the neck.

A poultice of hot Antiphlogistine over the nose and extending one inch on each side, applied before retiring, relieves "the stuffed up" feeling in a very short time. The pain subsides and the patient, breathing in a normal manner, usually has a restful sleep, and morning finds a considerable improvement with all of the distressing symptoms abated.

